# Marsupialization as an alternative to reconstruction in endoscopic skull base surgery. How I do it

**DOI:** 10.1007/s00701-024-06003-3

**Published:** 2024-02-22

**Authors:** Luca Ferlendis, Bianca Bossi, Paolo Castelnuovo, Davide Locatelli

**Affiliations:** 1 Division of Neurosurgery, Department of Biotechnology and Life Sciences, University of Insubria, Ospedale Di Circolo E Fondazione Macchi, 21100 Varese, Italy; 2 Division of Otorhinolaryngology, Department of Biotechnology and Life Sciences, University of Insubria, Ospedale Di Circolo E Fondazione Macchi, 21100 Varese, Italy

**Keywords:** Nasoseptal flap, Marsupialization, Endoscopic skull base surgery, Intracranial lesions, Skull base reconstruction, Alternative

## Abstract

**Background:**

To treat extradural solid-cystic lesions of the ventral skull base, a pedicled nasoseptal flap (NSF) maintains patency of the marsupialized cavity and prevents restenosis and cyst recurrence.

**Methods:**

The authors provide a step-by-step description of the surgical technique valid in different lesions of the skull base, all treated via the endoscopic endonasal approach (EEA). The application is demonstrated by an operative video.

**Conclusion:**

In selected lesions, endoscopic marsupialization using an NSF ensures drainage and ventilation of the surgical cavity. Re-epithelialization provided by a pedicled flap is a viable alternative to multilayer skull base reconstruction.

**Supplementary Information:**

The online version contains supplementary material available at 10.1007/s00701-024-06003-3.

## Introduction

Endoscopic endonasal approaches are a well-established surgical technique in the treatment of ventral skull base pathologies. EEAs improved greatly in the last 20 years due to the development of dedicated corridors, reconstructive techniques, and increasingly modern instruments. In our experience, we have demonstrated how to use the pedicled nasoseptal flap in conjunction with marsupialization of expansive cystic (cholesterol granulomas) [4] or inflammatory (mucoceles) [9] lesions for therapeutic purposes. With this technique, patency of the operative cavity is therefore achieved, re-epithelialization is encouraged, and recurrences caused by scarring are prevented [5]. Once re-epithelialization of the surgical cavity is accomplished, complete closure of the skull base is obtained.

In the past years, we have used this technique as a valid alternative to skull base reconstruction to prevent mucocele formation in the surgical neo-cavity in various skull base extradural lesions. Specifically, our case series in the last five years consists of 8 cholesterol granulomas (CGs), 2 trigeminal schwannomas, and 1 benign notochord cell tumor’s biopsy. The purpose of this work is to demonstrate and illustrate the effectiveness of this technique and to offer practical surgical guidance in the process.

## Relevant surgical anatomy

To reach the ventral skull base, a comprehensive knowledge of the anatomy from an endoscopic point of view is required. For a paraseptal transsphenoidal approach (TSA), [2] the sphenoidal sinus (SS) represents the epicenter of the surgical anatomy. The SS is divided by the intersphenoidal septum with different pneumatization patterns. The sellar floor is in the posterior wall of the sphenoidal sinus and continues above with the planum sphenoidalis and below with the clivus. Once an anterior sphenoidotomy is performed, the main anatomical landmarks that are fundamental to recognize are the optic nerve bulging above and the parasellar carotid artery prominence below. The lateral optico-carotideal recess lies between them and represents the pneumatization of the optic strut.

In a trans-ethmoidal-pterygo-sphenoidal approach (TEPS), [2, 4] the pterygopalatine fossa (PF) must be reached at the level of the sphenopalatine foramen by removing the orbital process of the palatine bone and the posterior wall of the maxillary sinus. Then, the vidian canal (VC) must be detected to locate the anterior genu of the horizontal segment of the petrous internal carotid artery (ICA) [6].

Using a transclival approach (TCA), [4, 3] the identification of the vidian nerve and the ICA marks the petroclival junction, which must be removed to gain access to the petrous apex.

## Description of the technique

### Operative setup

The patient is positioned supine in an anti-Trendelenburg position, with a mobile head slightly flexed. Depending on the site of the lesion and the individual anatomy, the following approaches can be employed: a TSA (to sellar/parasellar or clival region), a TEPS (lateral suprasellar region, pterygopalatine fossa, middle cranial fossa), or transclival (to petrous apex) approach. The required instrumentation is composed of an endoscopic endonasal set, a Neuronavigation system, an acoustic US Doppler, and adequate neurophysiological monitoring.

### Surgical procedure

A “two nostrils – four hands technique” [1] is adopted during every procedure to enlarge the surgical field, increasing the surgical effectiveness.

Regardless of the chosen EEA (TSA, TEPS, TCA), the key surgical step is to harvest the pedicled NSF. It is important to bear in mind that the NSF must be large enough to cover at least 180 degrees of the circumference of the neo-cavity to ensure re-epithelialization [4].

The NSF is vascularized by the septal branches of the sphenopalatine artery (SPA), and the harvesting technique may vary depending on the approach or the site of the lesion. These techniques are well described in the literature; therefore, they will not be discussed in this article in detail [7, 8].

In brief, a rescue NSF (RNSF) [7] is preferred for TSA, whereas a modified RNSF [8] is chosen in case of a TCA. If a TEPS is needed, the ipsilateral flap is created below the tail of the middle turbinate following the choana and extending through the vomer to the maxillary crest as already described [4]. Once the pterygopalatine fossa is reached, the flap is then stored in the maxillary sinus to be protected during surgery.

#### Placement of the NSF in the cavity

**Fig. 1:**
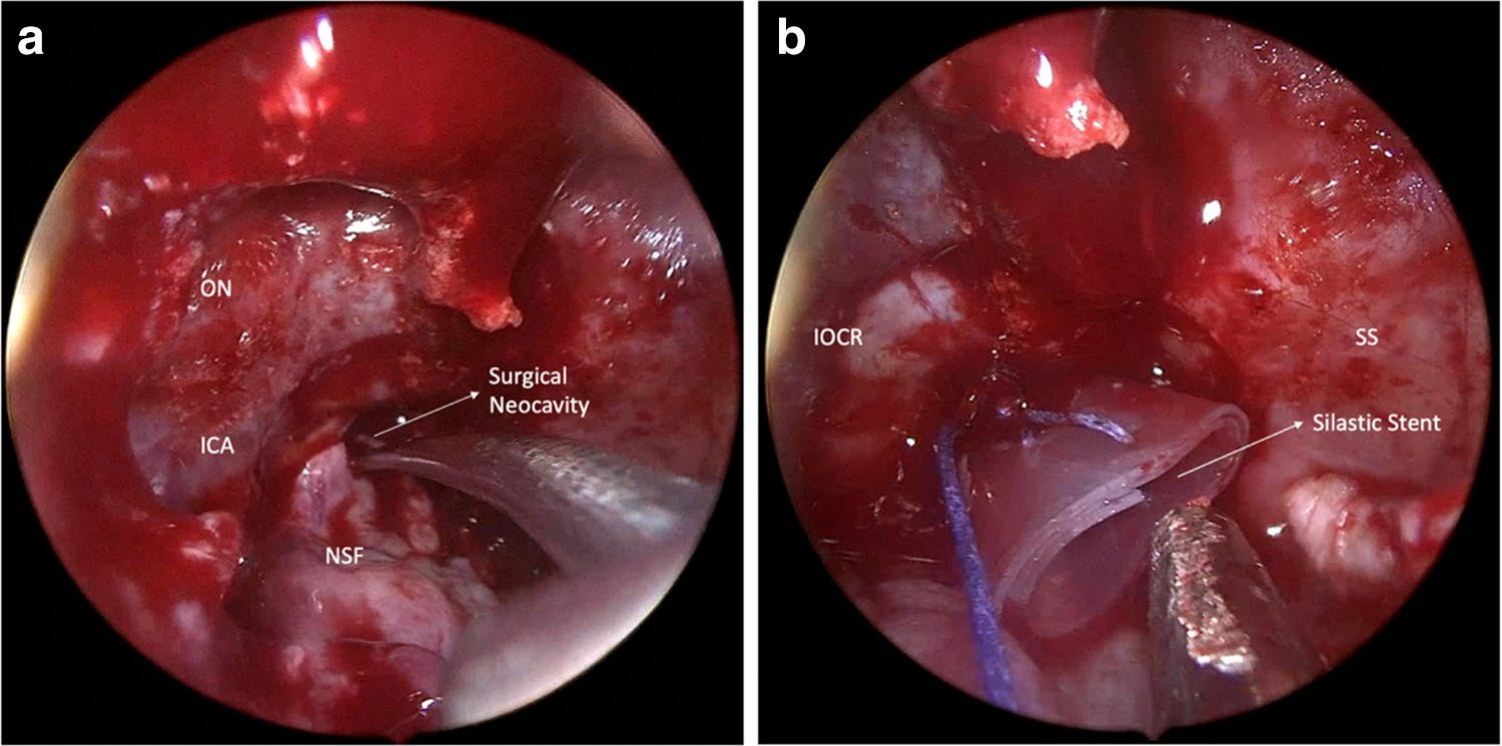
The nasospetal flap (NSF) is placed in the marsupialized surgical cavity (**a**) and then is stabilized with a silastic stent (**b**), which will remain on site for two weeks. ON: optic nerve; IOCR: interoptical carotid recess

The NSF is inserted into the cavity once the asportation/biopsy is accomplished. To gain the rotational degree of the flap, it is always necessary to drill the basisphenoid to increase its length and avoid constricting the vascular supply. The coverage of the marsupialized cavity’s margins must extend for at least half of its perimeter. The flap is placed over the denuded bone after the sphenoidal mucosa had been moved, covering its surface to promote proper integration and re-epithelization. A soft silastic stent is used to stabilize the flap in the cavity, and it is left in place for two weeks (Fig. 1). For good hemostatic control, endonasal swabs are placed in each nostril.

### Postoperative course

**Fig. 2:**
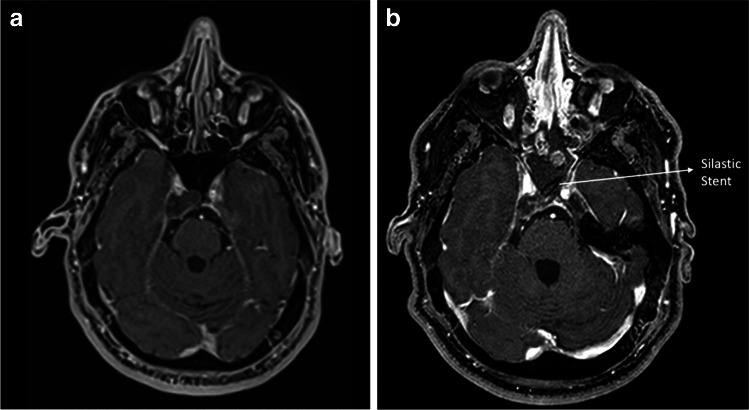
Preoperative (**a**) and postoperative (**b**) MRI of a patient undergoing endoscopic marsupialization of a biopsied right parasellar lesion

Each patient undergoes a postoperative encephalic CT scan (or MRI) on the first postoperative day (Fig. 2). After 24–48 h, the first endonasal endoscopic medication is performed and tampons are removed. After two weeks, the silastic stent is then removed, and the endoscopic examinations will follow at 40-, 60-, and 120-day post-surgery. Follow-up is tailored according to the outcome of the histologic examination.

## Indications

**Fig. 3:**
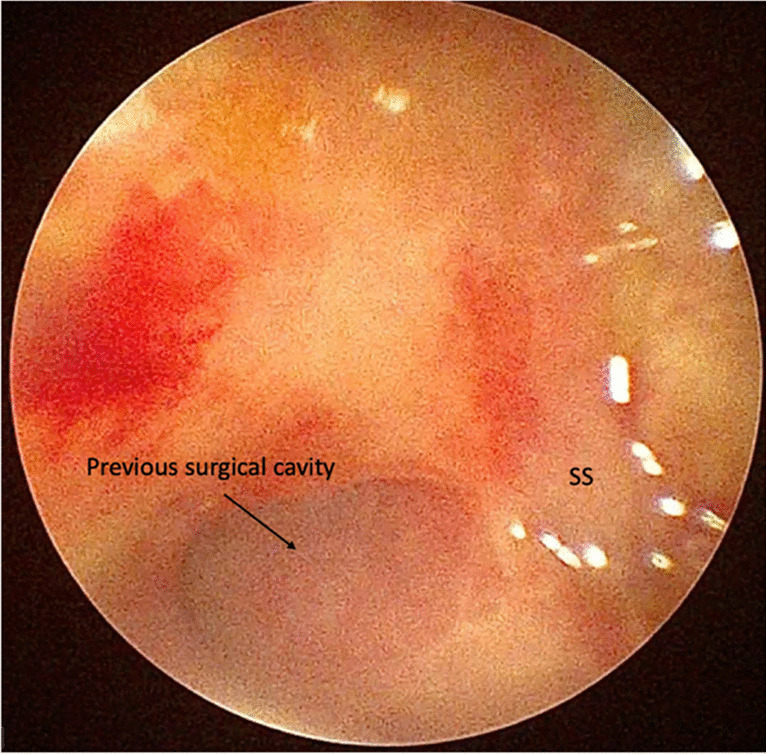
At endoscopic inspection after one month, initial re-epithelialization can be seen

**Fig. 4:**
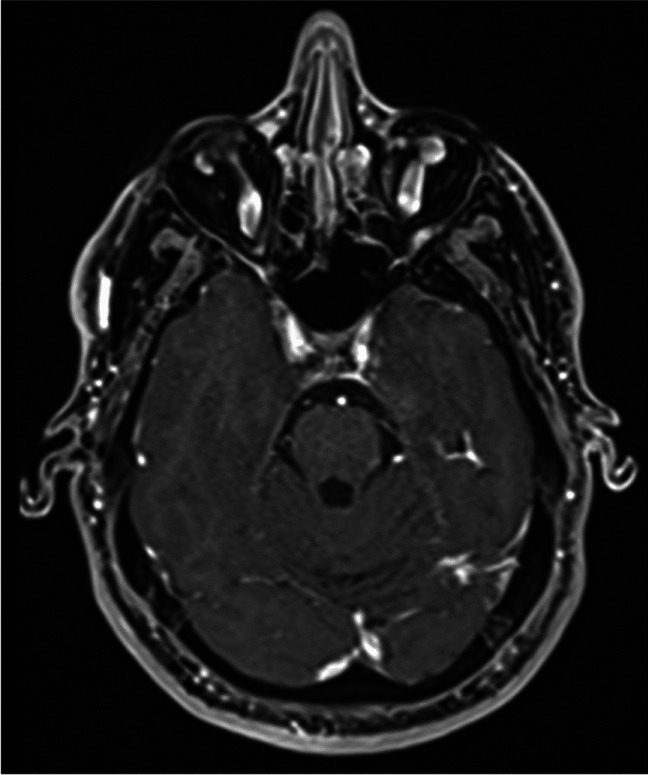
Follow-up MRI at six months showing complete asportation of the tumor and re-epithelization of the skull base

EEA are minimally invasive and effective techniques for the management of off-midline pathologies. As we use the pedicled NSF in the endoscopic treatment of CGs, we extend this technique to the management of other pathologies. According to our experience, endoscopic marsupialization using an NSF is indicated only in extradural lesions with a solid-cystic component and in those cases in which the absence of intraoperative CSF leakage can be established. When re-epithelialization of the surgical cavity has been completed, this becomes part of the nasal cavities, and a full closing of the skull base is achieved (Figs. 3 and 4).

## Limitations

This technique cannot be used in the case of intradural lesions or case of intraoperative evidence of CSF leakage.

## How to avoid complications

A comprehensive experience in endoscopic basic skull base surgery (ESBS) is the first key to performing advanced approaches to ventral skull base pathologies. Proper planning of surgery by analysis of neuroimaging examinations is essential as well. During surgery, the assistance of neuronavigation, the proper endoscopic set, the US Doppler, and an experienced team are basic elements that grant to minimize complications.

Close clinical monitoring and endoscopic examinations are mandatory in the immediate postoperative period to rule out signs of endocranial hypotension or CSF leakage. In the postoperative period, the patient is discharged with the above-described follow-up, which is essential to monitor the normal evolution of the surgical field.

## Specific information for the patient

Patients undergoing ESBS should always be informed about advantages and possible complications. Among the most common postoperative complications we need to mention are CSF leak and nasal morbidities (such as olfaction deficit). On the contrary, the most dangerous and devastating complication that can occur intraoperatively is ICA injury, which fortunately in literature is reported in a very small percentage of cases (incidence of 0.016 to 1%) [10].

## Supplementary Information

Below is the link to the electronic supplementary material.

Supplementary file1 (MP4 337155 KB)

## Data Availability

Not applicable.
